# Incorporation of protein induced by vitamin K absence or antagonist-II into transplant criteria expands beneficiaries of liver transplantation for hepatocellular carcinoma: a multicenter retrospective cohort study in China

**DOI:** 10.1097/JS9.0000000000000729

**Published:** 2023-11-20

**Authors:** Kai Wang, Libin Dong, Qian Lu, Zhe Yang, Xiaoli Fan, Fengqiang Gao, Wenwen Ge, Zhoucheng Wang, Zhisheng Zhou, Di Lu, Xuyong Wei, Qiang Wei, Li Zhuang, Lunxiu Qin, Qifa Ye, Jiayin Yang, Jiahong Dong, Shusen Zheng, Xiao Xu

**Affiliations:** a Zhejiang University School of Medicine, Hangzhou; b Key Laboratory of Integrated Oncology and Intelligent Medicine of Zhejiang Province, Hangzhou; c Institute of Organ Transplantation, Zhejiang University, Hangzhou; d Center of Hepatobiliary Pancreatic Disease, Beijing Tsinghua Changgung Hospital, School of Clinical Medicine, Tsinghua University, Beijing; e Department of Hepatobiliary and Pancreatic Surgery, Shulan Hospital of Hangzhou, Hangzhou; f Zhongnan Hospital of Wuhan University, Institute of Hepatobiliary Diseases of Wuhan University, Transplant Center of Wuhan University, Wuhan; g National Center for Healthcare Quality Management in Liver Transplant, Hangzhou; h Department of General Surgery, Huashan Hospital, Cancer Metastasis Institute, Fudan University, Shanghai; i Department of Liver Surgery and Liver Transplantation Center, West China Hospital of Sichuan University, Chengdu; j Department of Hepatobiliary and Pancreatic Surgery, the First Affiliated Hospital, Zhejiang University School of Medicine, Hangzhou; k NHC Key Laboratory of Combined Multi-organ Transplantation, Hangzhou, China.

**Keywords:** hepatocellular carcinoma, liver transplantation, prognostic prediction, protein induced by vitamin K absence or antagonist-II, α-fetoprotein

## Abstract

**Introduction:**

In order to maximize the utilization of precious donor liver, precisely determining potential hepatocellular carcinoma (HCC) candidates who will benefit from liver transplantation (LT) is essential. As a crucial diagnostic biomarker for HCC, protein induced by vitamin K absence or antagonist-II (PIVKA-II) has become one of the key indicators for assessing tumor recurrence risk after LT. This study aims to investigate the role of PIVKA-II in recipient selection and prognostic stratification.

**Methods:**

The clinicopathologic data of HCC patients undergoing LT from 2015 to 2020 in six Chinese transplant centers were collected. Univariate and multivariate analyses were performed to determine risk factors for disease free survival (DFS). Based on these risk factors, survival analysis was made by Kaplan–Meier method and their value in prognostic stratification was assessed.

**Results:**

A total of 522 eligible HCC patients with pre-LT PIVKA-II records were finally included in this study. Tumor burden>8 cm, α-fetoprotein>400 ng/ml, histopathologic grade III and PIVKA-II>240 mAU/ml were identified as independent risk factors for DFS. DFS of patients with PIVKA-II≤240 mAU/ml (*N*=288) were significantly higher than those with PIVKA-II>240 mAU/ml (*N*=234) (1-year, 3-year, and 5-year DFS: 83.2, 77.3, and 75.9% vs. 75.1, 58.5, and 50.5%; *P*<0.001). Compared with Hangzhou criteria (*N*=305), incorporating PIVKA-II into Hangzhou criteria (including tumor burden, α-fetoprotein, and histopathologic grade) increased the number of patients with eligibility for LT by 21.6% but achieved comparable DFS and overall survival.

**Conclusions:**

Incorporating PIVKA-II into existing LT criteria could increase the number of eligible HCC patients without compromising post-LT outcomes.

## Introduction

Highlights

- This is the first multicenter study to investigate the role of protein induced by vitamin K absence or antagonist-II (PIVKA-II) in hepatocellular carcinoma (HCC) recipient selection and prognostic stratification based on a Chinese Liver transplantation (LT) population.
- Preoperative PIVKA-II correlates with outcome of LT recipients with HCC.
- Incorporating PIVKA-II into existing LT criteria could increase access to LT for HCC candidates without compromising postoperative outcomes.

Hepatocellular carcinoma (HCC) is one of the most aggressive and lethal malignancies worldwide, with a dismal prognosis and a 5-year survival rate of less than 20%^1,2^. In China, HCC is the first leading cause of death among various types of cancer in male population aged less than 60^3^. Recent advances in therapy for HCC have not substantially improved patient survival^4^. Liver transplantation (LT) is still an optimal curative approach for selected patients with HCC. To avoid tumor relapse after LT, Mazzaferro *et al*.^5^ proposed Milan criteria in 1996. For more than two decades, the Milan criteria stood the test of time and represented a benchmark for guiding the selection of HCC candidates for LT^6^. Although the Milan criteria could achieve a favorable long-term outcome, it has been challenged over recent years due to restricted limitations in recipient selection, resulting in decreased access to LT for HCC patients. Ideally, such a selection tool should take into consideration of HCC biological behavior, a characteristic that is not measurable by conventional imaging examination. As a key biomarker in HCC detection, α-fetoprotein (AFP) reflects biological features of HCC and is related to recipients’ long-term survival after LT^7–9^. Therefore, several modified selection criteria based on tumor size and preoperative AFP have been proposed such as AFP model^10^ and Hangzhou criteria^11^. The AFP model exhibited better predictive performance in post-LT tumor recurrence than the Milan criteria in hepatitis C virus-related HCC patients and has been applied to select HCC candidates for LT by the Liver Transplantation French Study Group for organ sharing^12^. In a multicenter study with a cohort of 6012 HCC cases, Xu *et al*.^11^ reported that Hangzhou criteria provided a good prognosis and an expansion of 51.5% in LT eligibility compared with the Milan criteria. Given the high heterogeneity of HCC, additional biomarkers could reflect tumor progression and malignancy more comprehensively^13,14^.

Protein induced by vitamin K absence or antagonist-II (PIVKA-II), an immature prothrombin with insufficient coagulation activity, has been widely applied for clinical HCC diagnosis^15–17^. The excessive production of PIVKA-II predicts aggressive tumor behavior including vascular invasion and HCC metastasis^18–22^. Thus, monitoring PIVKA-II in HCC patients might serve as a valuable reference for instituting a personalized treatment program^23^. Lee *et al*.^24^ demonstrated that preoperative PIVKA-II could be used to assess the outcomes of living donor liver transplantation (LDLT). However, this conclusion may not be suitable for the Chinese population due to the heterogeneity of the mainstream type of LT. Deceased donor liver transplantation (DDLT) is a more common operation than LDLT for HCC in China. Previous studies reported that LDLT is associated with a higher incidence of tumor relapse than DDLT, which affected the robustness of the results.

The aim of the present study was therefore to further evaluate the role of PIVKA-II in recipient selection and prognostic stratification in a multicenter Chinese cohort.

## Materials and methods

### Patients

**Figure 1:**
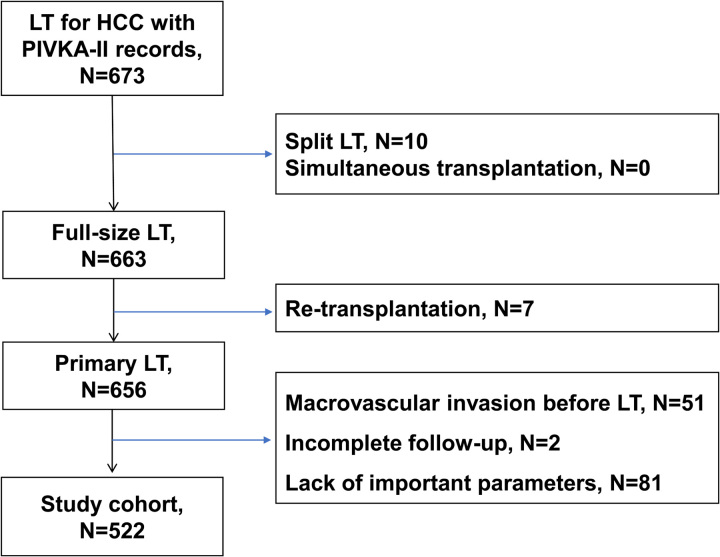
Flowchart for patient enrollment. HCC, hepatocellular carcinoma; LT, liver transplantation.

The present retrospective study included HCC patients who underwent LT between January 2015 and December 2020 from six transplant centers in China. Clinicopathological data of patients were extracted from the prospectively maintained China Liver Transplant Registry (CLTR) database. The patient selection process is depicted in Figure 1. From January 2015 to December 2020, 673 HCC patients received LT with preoperative PIVKA-II records in the six transplant centers. The assay kits for PIVKA-II detection of each center included Picolumi PIVKA-II kit (Lumipulse G1200 automated immunoassay instrument, Fujirebio Inc.), and ARCHITECT PIVKA-II Reagent Kit (ARCHITECT i2000SR automatic analyzer, Abbott Diagnostics).

HCC was diagnosed according to the current guidelines of American Association for the Study of Liver Diseases (AASLD)^25^. The exclusion criteria were as follows: (1) patients who received split LT or simultaneous transplantation, (2) patients who received retransplantation, (3) patients with macroscopic portal vein tumor thrombosis or other macrovascular invasion, (4) patients with incomplete follow-up or incomplete important parameters records. After applying the exclusion criteria, a total of 522 patients were included in the analysis. No liver grafts from prisoners were used for LT. All HCC candidates in the waiting list for LT were assessed preoperatively using abdominal ultrasound, computed tomography (CT), MRI, positron emission tomography-CT, bone scintigraphy, and colonoscopy to rule out extrahepatic metastasis, which was a contraindication for LT. Post-LT follow-ups were carried out regularly for all recipients after discharge. Patients who did not attend follow-up appointments were contacted by study nurses at the local transplant center via telephone. Liver function tests, serum biomarker examination, chest CT, abdominal ultrasound, abdominal CT, and bone scans were routinely performed every 6 months for the first 2 or 3 years and annually thereafter. Treatments for tumor recurrence after transplant were carried out in reference to the Chinese clinical practice guideline on LT for HCC^26,27^. The therapeutic schedule was adjusted according to the actual condition of patients and the usual practice of each center. According to the clinical guidelines, surgical resection was recommended as the primary treatment option for resectable recurrent lesions. In cases where surgical resection was not feasible, individualized approaches were selected to prolong the survival of patients. Transarterial chemoembolization (TACE) and/or local ablation [e.g. radiofrequency ablation (RFA)] were proposed for patients with unresectable intrahepatic recurrence. External beam radiotherapy was used in case of bone metastasis. Systemic chemotherapy and/or molecular targeted therapy (e.g. sorafenib, regorafenib, and lenvatinib) was administered for multifocal intrahepatic and extrahepatic recurrence. Individualized treatment regimens for tumor recurrence were also suggested by the European Association for the Study of the Liver clinical practice guideline^28^. This retrospective study has been reported in line with Strengthening The Reporting Of Cohort Studies in Surgery (STROCSS) guidelines^29^ (Supplemental Digital Content 1, http://links.lww.com/JS9/B360). Our study was conducted in accordance with the Declaration of Helsinki and approved by the CLTR. Informed consent was waived as previously collected data that did not include personally identifiable information were used.

### Data collection

Baseline clinicopathologic data were recorded: age, sex, BMI, hepatic cirrhosis, hepatitis B virus (HBV) infection, AFP, PIVKA-II, preoperative loco-regional therapy, preoperative hepatic resection, model of end-stage liver disease (MELD) score, Child-Pugh classification, ascites, hepatic encephalopathy, preoperative hypertension, preoperative diabetes mellitus, cold ischemia time, warm ischemia time, ABO compatibility, tumor burden, and histopathologic grade of tumor differentiation. Tumor burden represents the sum of tumor lesion diameters. The histopathologic grade of tumor differentiation was determined by the Edmondson and Steiner grading system. Preoperative loco-regional therapy, including RFA and TACE, was performed to control or reduce the tumor lesion. The endpoints for the current analysis were patient death or tumor recurrence. Overall survival (OS) was defined as the duration from the date of LT to death from any cause. Disease free survival (DFS) was defined as the interval from the date of LT to tumor recurrence, or death from any cause. Recipients were considered censored on the last follow-up date if still alive.

### Statistical analysis

Continuous variables were expressed as median and interquartile range (IQR). Categorical variables were expressed as number and percentages. Receiver operating characteristic (ROC) curves were constructed and the optimal PIVKA-II cut-off value in terms of predicting tumor recurrence was determined via the maximum Youden index (sensitivity + specificity - 1) for subsequent analysis. Univariable and multivariable Cox proportional hazard regression models were carried out to identify the risk factors for DFS. Only factors with *P*<0.05 in the univariate analysis were then incorporated into the multivariate analysis. OS and DFS were calculated using Kaplan–Meier method. For survival analysis, a log-rank test was used to assess group differences. Statistical analysis was performed with R software (R version 4.0.5).

## Results

### Baseline characteristics of the cohorts

The current study included a total of 522 recipients. The median follow-up time was 29.8 months with IQR of 27.2 and 31.7 months. Preoperative baseline information of the HCC patients was shown in Table 1. The median age was 53.6 years (IQR 47.4–60.4) and 468 (89.7%) recipients were male. In the whole cohort, 202 (38.7%) fulfilled Milan criteria and 305 (58.4%) fulfilled Hangzhou criteria. Median pre-LT serum PIVKA-II was 143.7 mAU/ml (IQR 33.0–1401.0), median pre-LT serum AFP was 25.9 ng/ml (IQR 5.4–149.8). The number of recipients with Child-Pugh class A was 137 (26.2%), Child-Pugh class B was 166 (31.8%), and Child-Pugh class C was 219 (42.0%). There were 239 (45.8%) patients receiving pre-LT TACE, 85 (16.3%) patients receiving pre-LT RFA, and 122 (23.4%) patients receiving pre-LT hepatic resection.

**Table 1:** Clinical characteristics of the study participants.

Characteristic	Levels	Value
Sex	Male	468 (89.7%)
	Female	54 (10.3%)
Age, years	Median (IQR)	53.6 (47.4–60.4)
BMI, kg/m^2^	Median (IQR)	23.5 (21.6–25.7)
Hepatic cirrhosis	Absent	69 (13.2%)
	Present	453 (86.8%)
HBV infection	Absent	51 (9.8%)
	Present	471 (90.2%)
MELD	≤20	172 (33.0%)
	>20	350 (67.0%)
Child-Pugh stage	A	137 (26.2%)
	B	166 (31.8%)
	C	219 (42.0%)
Ascites	Absent	161 (30.8%)
	Slight/Moderate	268 (51.3%)
	Severe	93 (17.8%)
Hepatic encephalopathy	Absent	449 (86.0%)
	Slight/Moderate	67 (12.8%)
	Severe	6 (1.1%)
Hypertension	Absent	413 (79.1%)
	Present	109 (20.9%)
Diabetes mellitus	Absent	433 (83.0%)
	Present	89 (17.0%)
AFP, ng/ml	Median (IQR)	25.9 (5.4–149.8)
PIVKA-II, mAU/ml	Median (IQR)	143.7 (33.0–1401.0)
Tumor number	1	251 (48.1%)
	≥2	271 (51.9%)
Tumor burden, cm	≤8	274 (52.5%)
	>8	248 (47.5%)
Histopathologic grade	I/II	456 (87.4%)
	III	66 (12.6%)
Pre-LT TACE	Absent	283 (54.2%)
	Present	239 (45.8%)
Pre-LT RFA	Absent	437 (83.7%)
	Present	85 (16.3%)
Pre-LT hepatic resection	Absent	400 (76.6%)
	Present	122 (23.4%)
Milan criteria	Fulfilling	202 (38.7%)
	Exceeding	320 (61.3%)
Hangzhou criteria	Fulfilling	305 (58.4%)
	Exceeding	217 (41.6%)
Cold ischemia time, hour	≤8	210 (40.2%)
	>8	312 (59.8%)
Warm ischemia time, minute	≤10	415 (79.5%)
	>10	107 (20.5%)
Blood type match level	Match	506 (96.9%)
	Mismatch	16 (3.1%)

AFP, α-fetoprotein; HBV, hepatitis B virus; IQR, interquartile range; LT, liver transplantation; MELD, model for end-stage liver disease; PIVKA-II, protein induced by vitamin K absence or antagonist-II; RFA, radio frequency ablation; TACE, transcatheter arterial chemoembolization.

### ROC analysis for pre-LT PIVKA-II

**Figure 2:**
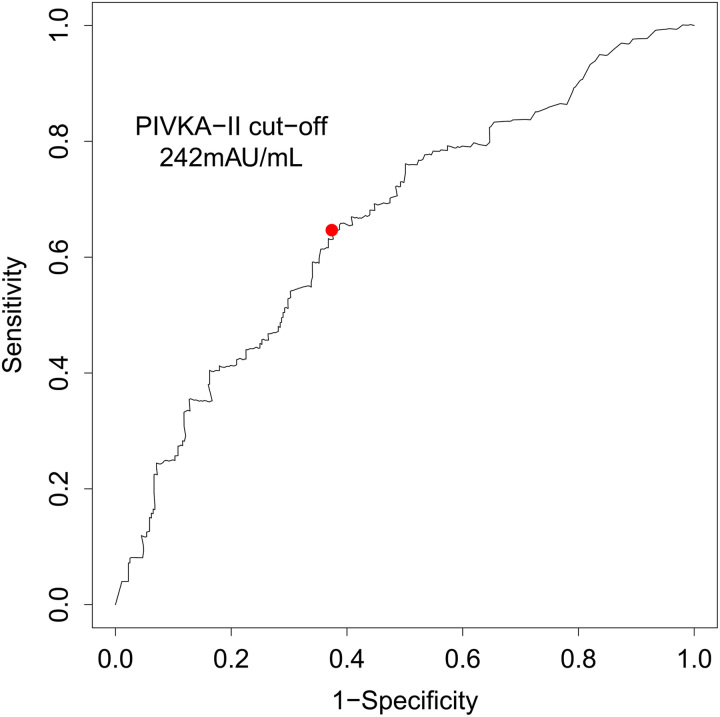
Receiver-operating characteristic curve showed optimal cut-point of PIVKA-II for tumor recurrence. PIVKA-II, protein induced by vitamin K absence or antagonist-II.

Post-LT tumor recurrence was observed in 65 (12.5%) patients. ROC curve analysis was carried out to determine the optimal pre-LT PIVKA-II cut-off value in terms of predicting post-LT recurrence (Fig. 2). A cut-off of 242 mAU/ml for PIVKA-II maximized the Youden index. For computational convenience, we considered PIVKA-II of 240 mAU/ml as the cut-off for subsequent analysis. In the whole population, there were 288 with PIVKA-II ≤240 mAU/ml (55.2%) and 234 with PIVKA-II >240 mAU/ml (44.8%).

### Univariate and multivariate analysis for DFS

Univariate analysis identified tumor burden>8 cm, pre-LT AFP >400 ng/ml, pre-LT PIVKA-II >240 mAU/ml, tumor histopathologic grade III, and present pre-LT TACE as the significant risk factors for DFS. Multivariate analysis showed that tumor burden >8 cm (HR=2.37; 95% CI: 1.564–3.593; *P*<0.001), pre-LT AFP >400 ng/ml (HR=1.897; 95% CI: 1.219–2.953; *P*=0.005), pre-LT PIVKA-II >240 mAU/ml (HR=1.558; 95% CI: 1.005–2.414; *P*=0.047), and tumor histopathologic grade III (HR=2.031; 95% CI: 1.291–3.194; *P*=0.002) were the independent risk factors for DFS (Table 2). Furthermore, we confirmed the impact of these independent risk factors on DFS and OS (Fig. 3A–D). The 1-year, 3-year, and 5-year DFS and OS for patients without these independent risk factors were significantly higher than those with independent risk factors.

**Table 2:** Univariable and multivariable Cox regression analyses of prognostic factors for disease free survival in all patients.

	Univariable		Multivariable	
Variable	HR (95% CI)	*P*	HR (95% CI)	*P*
Recipient sex, Female vs Male	1.041 (0.559–1.941)	0.899		
Recipient age, ≤60 vs >60 years	0.826 (0.529–1.290)	0.401		
Hepatic cirrhosis, Absent vs Present	1.108 (0.593–2.069)	0.748		
Diabetes mellitus, Absent vs Present	0.904 (0.539–1.516)	0.703		
Hypertension, Absent vs Present	0.920 (0.572–1.479)	0.730		
MELD score, ≤20 vs >20	1.352 (0.889–2.056)	0.158		
PIVKA-II, ≤240 vs >240 mAU/ml	2.596 (1.756–3.839)	<0.001	1.558 (1.005–2.414)	0.047
AFP, ≤400 vs >400 ng/ml	2.773 (1.822–4.220)	<0.001	1.897 (1.219–2.953)	0.005
Tumor burden, ≤8 vs >8 cm	3.351 (2.300–4.882)	<0.001	2.370 (1.564–3.593)	<0.001
Histopathologic grade, I/II vs III	3.010 (1.949–4.648)	<0.001	2.031 (1.291–3.194)	0.002
Pre-LT RFA, Absent vs Present	0.985 (0.588–1.652)	0.956		
Pre-LT TACE, Absent vs Present	1.675 (1.151–2.438)	0.007	1.379 (0.943–2.017)	0.097
Pre-LT hepatic resection, Absent vs Present	1.056 (0.684–1.629)	0.807		
Cold ischemia time, ≤8 vs >8 h	1.275 (0.856–1.898)	0.232		
Warm ischemia time, ≤10 vs >10 min	0.952 (0.568–1.596)	0.851		

AFP, α-fetoprotein; HR, hazard ratio; LT, liver transplantation; MELD, model for end-stage liver disease; PIVKA-II, protein induced by vitamin K absence or antagonist-II; RFA, radio frequency ablation; TACE, transcatheter arterial chemoembolization.

Figure 3Comparison of DFS and OS of patients in different risk groups. The DFS and OS curves for (A) Tumor burden ≤8 cm versus Tumor burden >8 cm, (B) Histopathologic grade I/II versus Histopathologic grade III, (C) AFP ≤400 ng/ml versus AFP >400 ng/ml (D) PIVKA-II ≤240 mAU/ml versus PIVKA-II >240 mAU/ml. AFP, α-fetoprotein; DFS, disease free survival; OS, overall survival; PIVKA-II, protein induced by vitamin K absence or antagonist-II.

**Figure:**
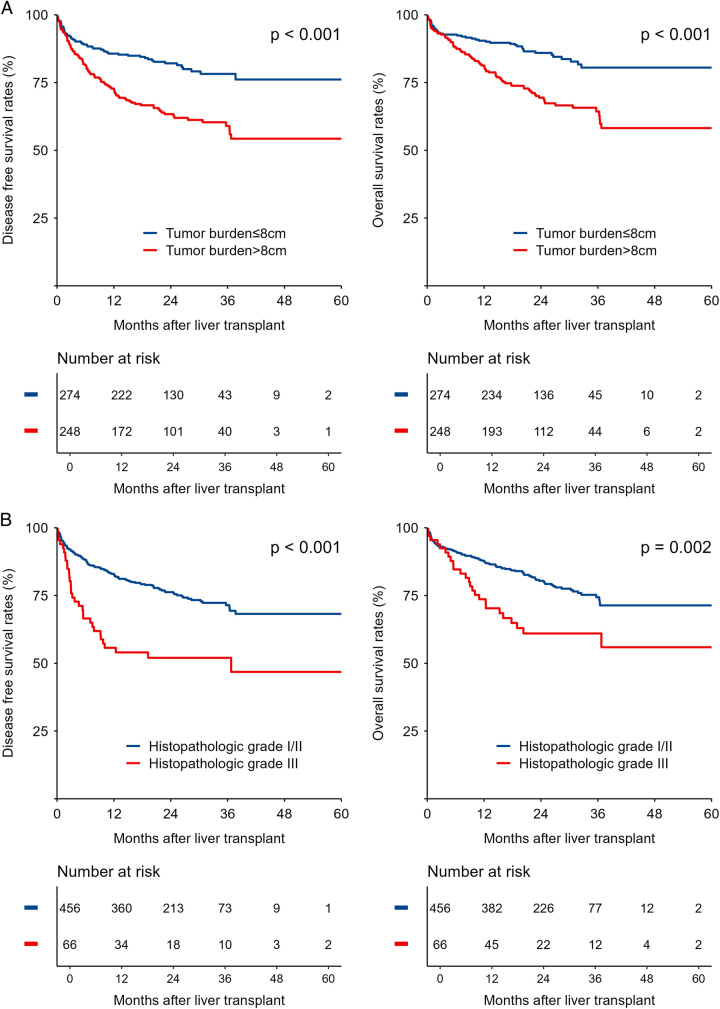

**Figure:**
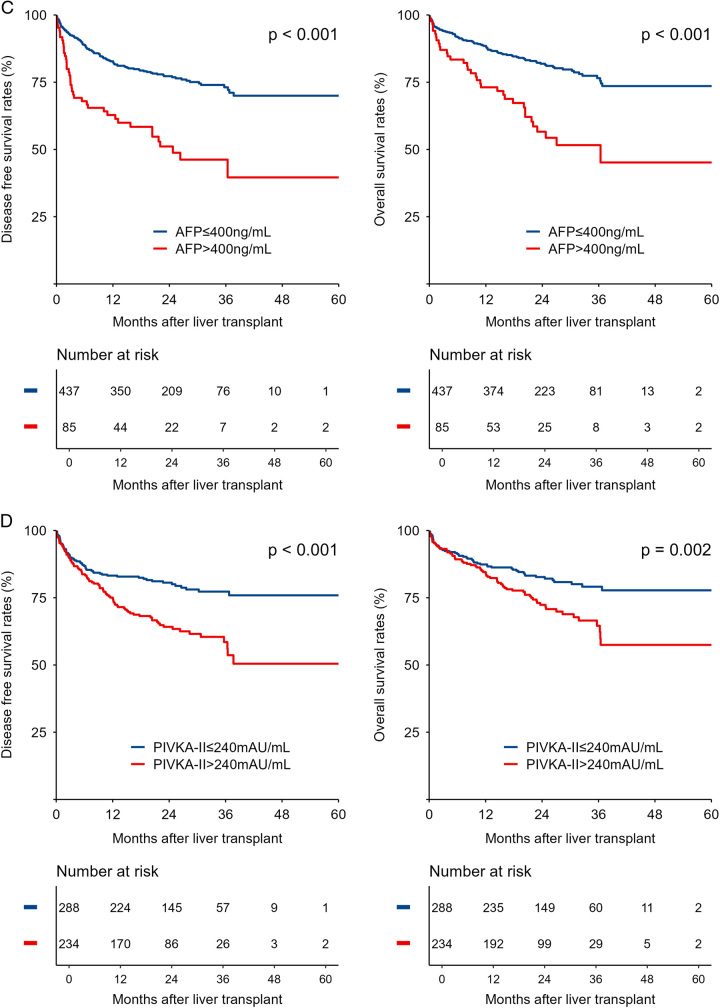

### Incorporate PIVKA-II into the Hangzhou criteria

**Figure 4:**
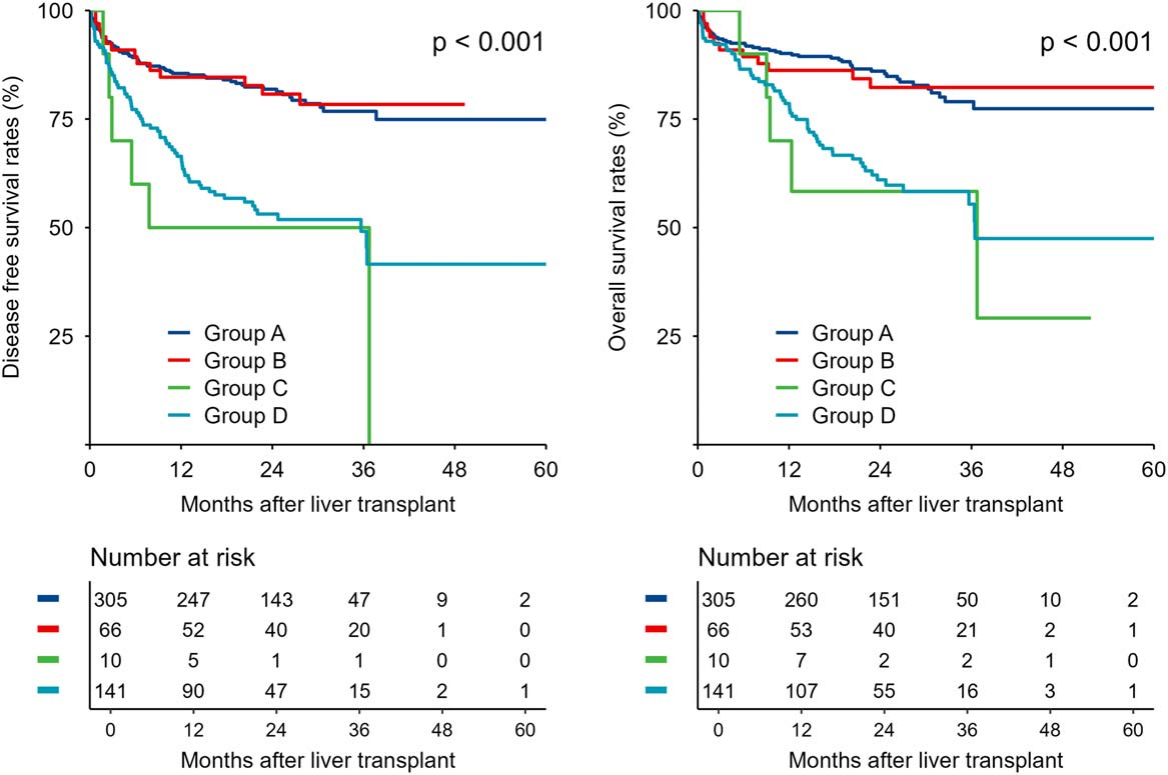
Comparison of DFS and OS of patients stratified by different risk factors. Patients were classified into four groups according to independent risk factors identified by multivariable analysis (Table 3). According to the cumulative survival curve, there was no significant difference between group A and group B (*P*=0.824). Similarly, no significant difference was observed between group C and group D (*P*=0.393). Patients of group A and group B had significantly improved prognosis compared with those of group C and group D. DFS, disease free survival; OS, overall survival.

The Hangzhou criteria was defined as follows: tumor burden ≤8 cm; or tumor burden >8 cm, with histopathologic grades I or II and pre-LT AFP ≤400 ng/ml, simultaneously^11^. Thus, the Hangzhou criteria has already included three risk factors (tumor burden, histopathologic grade, and pre-LT AFP) identified by multivariate analysis. Based on the results of the multivariate analysis and Hangzhou criteria, patients fell into four groups: (A) fulfilling the Hangzhou criteria as mentioned before (*N*=305, 58.4%); (B) tumor burden >8 cm, tumor histopathologic grade I/II, PIVKA-II ≤240 mAU/ml and AFP >400 ng/ml (*N*=66, 12.6%); (C) tumor burden >8 cm, tumor histopathologic grade III, PIVKA-II ≤240 mAU/ml and AFP ≤400 ng/ml (*N*=10, 1.9%); (D) other patients (*N*=141, 27.1%) (Table 3). According to the survival curve, there was no significant difference between group A and group B in DFS and OS (DFS: *P*=0.824; OS: *P*=0.920). Similarly, no significant difference was observed between group C and group D in DFS and OS (DFS: *P*=0.393; OS: *P*=0.534). Patients in group C and D had significantly reduced DFS and OS compared with those in group A and B and were deemed unsuitable for LT (*P*<0.001) (Fig. 4). Incorporation of PIVKA-II into Hangzhou criteria (HC&PIVKA-II) was made, as follows: (a) tumor burden ≤8 cm; (b) tumor burden >8 cm, but with histopathologic grade I/II and simultaneously at least 1 eligible tumor markers (PIVKA-II ≤240 mAU/ml or AFP ≤400 ng/ml) (Fig. S1, Supplemental Digital Content 2, http://links.lww.com/JS9/B361, which describes this prognostic stratification algorithm).

**Table 3:** Different risk groups of patients.

Group	Tumor burden ≤8 cm	PIVKA-II ≤240 mAU/ml	Histopathologic grade I/II	AFP ≤400 ng/ml
A	Yes	Yes/No	Yes/No	Yes/No
	No	Yes/No	Yes	Yes
B	No	Yes	Yes	No
C	No	Yes	No	Yes
D	Other patients			

AFP, α-fetoprotein; DFS, disease free survival; PIVKA-II, protein induced by vitamin K absence or antagonist-II.

### Comparisons of different transplant criteria

**Figure 5:**
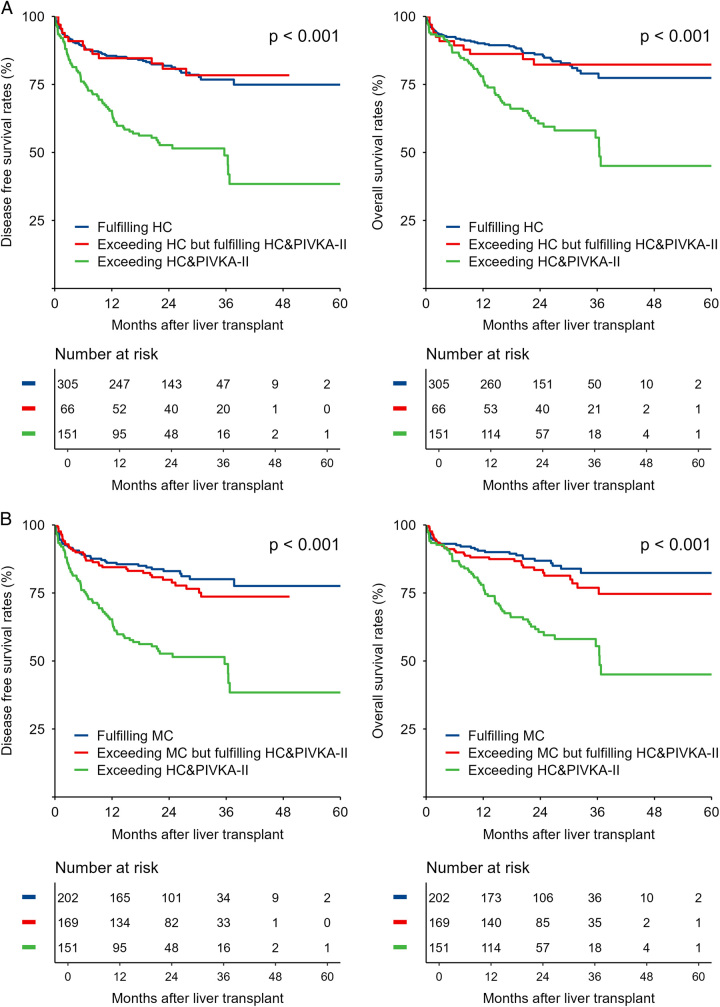
Comparison of DFS and OS of patients classified by Hangzhou criteria and HC&PIVKA-II. The DFS and OS curves for (A) Hangzhou criteria and HC&PIVKA-II, (B) Milan criteria and HC&PIVKA-II. DFS, disease free survival; HC, Hangzhou criteria;HC&PIVKA-II, incorporation of PIVKA-II into Hangzhou criteria; OS, overall survival; PIVKA-II, protein induced by vitamin K absence or antagonist-II.

Compared with the Hangzhou criteria, the HC&PIVKA-II increased the number of HCC candidates with eligibility for LT by 21.6% (*N*=66). The 1-year and 3-year DFS for the patients fulfilling the Hangzhou criteria (*N*=305) and those exceeding the Hangzhou criteria but fulfilling the HC&PIVKA-II (*N*=66) were 85.5 and 76.8% versus 84.6 and 78.4% (*P*=0.824). When comparing the OS for the patients fulfilling the Hangzhou criteria and those exceeding the Hangzhou criteria but fulfilling the HC&PIVKA-II, the 1-year, 3-year, and 5-year OS had no significant difference (90.1, 79.0, and 77.4% vs. 86.2, 82.3, and 82.3%) (*P*=0.920, Fig. 5A). Of those exceeding the HC&PIVKA-II (*N*=151), significant decreased DFS and OS were noted (*P*<0.001).

Compared with the Milan criteria, the HC&PIVKA-II increased the number of HCC candidates with eligibility for LT by 83.7% (*N*=169). No significant difference in DFS was observed between the patients fulfilling the Milan criteria (N=202) and those exceeding the Milan criteria but fulfilling HC &PIVKA-II (N=169) (1-year and 3-year DFS: 86.1 and 80.1% vs. 84.5 and 73.7%; *P*=0.402). Similarly, no significant difference in OS was observed between the patients fulfilling the Milan criteria and those exceeding the Milan criteria but fulfilling the HC&PIVKA-II (1-year, 3-year, and 5-year DFS: 90.5, 82.4, and 82.4% vs. 88.1, 76.9, and 74.7%; *P*=0.245, Fig. 5B). DFS and OS for the patients exceeding the HC&PIVKA-II were significantly worse than other patients (*P*<0.001). Overall, these results confirmed the feasibility of incorporating PIVKA-II into the current LT criteria for selecting eligible HCC candidates.

## Discussion

As a benchmark of LT recipient selection for HCC, the Milan criteria ensured significantly improved long-term outcomes. However, the Milan criteria showed to be too restrictive precluding access to LT for a substantial number of HCC patients, who might benefit from transplantation. A deep understanding of tumor biological behaviors and great efforts to increase access of HCC patients to LT led to the extended transplant criteria, which improved prognostic stratification^30^. To date, several transplant criteria or models with AFP have been established and validated. Xu *et al*. reported that incorporation of pre-LT AFP <400 ng/ml could increase the number of eligible HCC patients by 51.5% compared to the Milan criteria. Additionally, tumor recurrence in patients with AFP >100 ng/ml was significantly higher than those with AFP ≤100 ng/ml^11^. Due to the high heterogeneity of HCC, the incorporation of additional biomarkers holds the promise to expand and refine recipient selection further.

To our knowledge, this is the first multicenter study to investigate the role of PIVKA-II, a crucial biomarker of HCC widely used in Asian countries, in recipient selection and prognostic stratification based on a Chinese LT population. In this study, pre-LT PIVKA-II showed predictive power for tumor recurrence. ROC analysis defined 240 mAU/ml as the optimal cut-off value for PIVKA-II. Pre-LT PIVKA-II >240 mAU/ml was identified as an independent risk factor for DFS by multivariate Cox analysis and correlated with poor post-LT OS. It was reported that PIVKA-II was responsible for HCC progression through enhancing cell proliferation, extracellular matrix synthesis, and tumor angiogenesis in vivo and in vitro. A French study including 128 cases showed that patients with PIVKA-II >90 mAU/ml had 3.5 times the risk of MVI presence compared to those with PIVKA-II ≤90 mAU/ml^22^. Previous clinical studies also demonstrated that preoperative PIVKA-II was closely associated with tumor recurrence. Wang *et al*.^31^ found a significant difference in 2-year tumor recurrence after hepatectomy between patients with preoperative PIVKA-II ≥40 mAU/ml and those with PIVKA-II <40 mAU/ml (58.8 vs. 28.0%, *P*<0.001). In a retrospective, multicenter study, a risk score based on PIVKA-II and AFP could reflect tumor aggressiveness, and a high score of greater than 68 was determined as an independent prognostic factor of recurrence-free survival after RFA (HR=4.84; 95% CI: 1.83–35.68; *P*=0.008)^32^. Therefore, PIVKA-II has the potential to function as a favorable risk factor to be incorporated into existing transplant criteria. Several efforts have been made to implement this strategy^21,33^. Kim *et al*.^21^ reported that patients exceeding the existing LT criteria (Milan criteria, UCSF criteria, and Asan criteria) but fulfilling the criteria of AFP <150 ng/ml and PIVKA-II <100 mAU/ml, could achieve a favorable 5-year OS range from 65.2 to 74.5%. Besides, due to the variation of tumor features over time, the waiting duration of patients on the transplant list is a key parameter affecting post-LT prognosis. The waiting duration on the transplant list has gained increased attention, affecting the organ allocation policy in the USA^34,35^. Thus, to accurately evaluate the relationship between tumor features and post-LT prognosis, we selected the baseline of tumor at the most recent timepoint before LT.

In the present study, multiple tumor biological characteristics (PIVKA-II, AFP, and tumor histopathologic grade) and a traditional tumor morphological parameter were combined to stratify the outcomes of HCC patients after LT. The novel stratification approach defined a population including more HCC patients with equivalent outcomes compared with the Hangzhou criteria and the Milan criteria, suggesting the potential feasibility of performing LT in those population. Of note, China bore the greatest HCC burden globally. In 2020, it was reported that almost half of the world’s estimated cases and deaths from liver cancer occurred in China (45.3 and 47.1%, respectively)^36^. A huge number of HCC patients in China have already exceeded the Milan criteria at the time of diagnosis. Therefore, expanded LT criteria with novel perspectives is urgently needed to identify HCC patients with a low risk of tumor recurrence after LT. More importantly, medical insurance policies could be revised based on feasibility and scientificity of extended LT criteria in some Chinese provinces. In this regard, more patients with relatively late-stage HCC are more likely to be covered by the medical insurance and have less financial burden from LT.

There were also several limitations in this study. Firstly, not all Chinese transplant centers performed the PIVKA-II test for HCC patients before 2019. However, increased emphasis has been placed on the predictive value of PIVKA-II before LT, leading to more and more available PIVKA-II records. Thus, further studies with larger cohorts are warranted to validate and confirm these results in the future. Secondly, vitamin K deficiency or taking vitamin K antagonist drugs would cause elevated serum PIVKA-II^37^. This impact on the present results was not evaluated and may overestimate the cut-off value for PIVKA-II.

In conclusion, incorporating PIVKA-II into the Hangzhou criteria could act as an effective decision-making algorithm, leading to an increased number of HCC patients with eligibility for LT by 21.6% compared with the Hangzhou criteria without compromising post-LT outcomes. Therefore, this prognostic stratification, the HC&PIVKA-II, holds the promise to fulfill the recommendations of the Chinese LT society and guide the clinical management of LT for HCC.

## Ethical approval

Ethical approval for this study (Reference number: NO. 20220025) was provided by the Institutional Review Board of the China Liver Transplantation Registration Scientific Committee, Hangzhou, People’s Republic of China on 27 August 2022.

## Consent

Informed consent for publication was obtained from all authors.

## Sources of funding

This work was supported by Youth Program of National Natural Science Foundation of China (82003248); Key Program of National Natural Science Foundation of China (81930016); National Key Research and Development Program of China (2021YFA1100500); Major Research Plan of the National Natural Science Foundation of China (92159202).

## Author contribution

X.X., S.Z., J.D., and J.Y.: designed the research; K.W., L.D., Q.L. and Z.Y.: collected and organized data; K.W., L.D., X.F., F.G., W.G., Z.W., and Z.Z.: analyzed the data; K.W., L.D., Q.L., and Z.Y.: drafted the manuscript; D.L., X.W., Q.W., L.Z., L.Q., Q.Y., X.X., S.Z., J.D., and J.Y.: contributed to the critical revision of the manuscript. All authors contributed to the manuscript and approved the submitted version.

## Conflicts of interest disclosure

The authors of this manuscript have no conflicts of interest to disclose.

## Research registration unique identifying number (UIN)

1. Name of the registry: Clinical Trials.
2. Unique identifying number or registration ID: NCT05907772.
3. Hyperlink to your specific registration (must be publicly accessible and will be checked): https://clinicaltrials.gov/ct2/show/NCT05907772.

## Guarantor

Xiao Xu.

## Data availability statements

The data that support the findings of this study are available from the China Liver Transplant Registry but restrictions apply to the availability of these data, which were used under license for the current study, and so are not publicly available. Data are however available from the authors upon reasonable request and with permission of the China Liver Transplant Registry.

## Provenance and peer review

Not commissioned, externally peer-reviewed.

## Supplementary Material

SUPPLEMENTARY MATERIAL
