# Supplementary material for: Incorporation of protein induced by vitamin K absence or antagonist-II into transplant criteria expands beneficiaries of liver transplantation for hepatocellular carcinoma: a multicenter retrospective cohort study in China
Source: Int J Surg. 2023 Nov 20;109(12):4135–44. doi: 10.1097/JS9.0000000000000729 (PMC10720805; doi:10.1097/JS9.0000000000000729)

Title: Incorporation of protein induced by vitamin K absence or antagonist-II into transplant criteria expands beneficiaries of liver transplantation for hepatocellular carcinoma: A multi-center retrospective cohort study in China

Supplemental Digital Content

Fig. S1. The description of the stratification algorithm based on HC&PIVKA-II. HC&PIVKA-II: incorporation of PIVKA-II into Hangzhou criteria; AFP: α-fetoprotein; PIVKA-II: protein induced by vitamin K absence or antagonist-II.

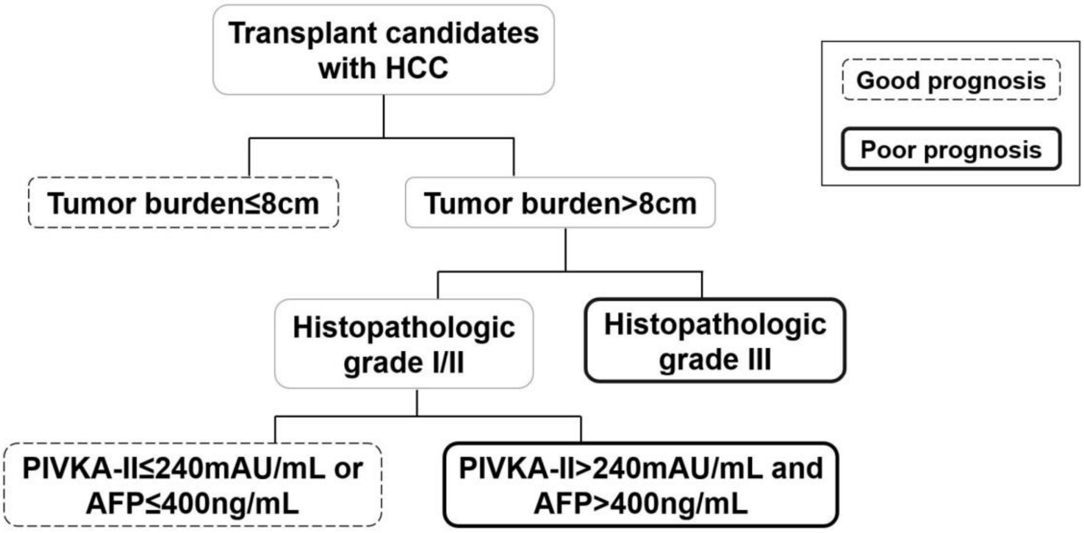

Supplement: SUPPLEMENTARY MATERIAL [file js9-109-4135-s002.pdf]
